# You never narc alone! A transpersonal model of personality and personality pathology, exemplified in the case of narcissism

**DOI:** 10.1177/09593543251415181

**Published:** 2026-01-26

**Authors:** Kerrin A. Jacobs, Emanuel Jauk

**Affiliations:** 1 University of Witten/Herdecke, Germany; 2 Medical School Berlin, Germany; 3 Medical University of Graz, Austria

**Keywords:** interpersonal theory, narcissism, personality, script theory, transpersonal pathology

## Abstract

Recent advances in the field of personality pathology have increasingly emphasized the relational and interpersonal dimensions of personality disorders. However, these frameworks often fall short in capturing broader, systemic dynamics that transcend the individual level. Taking the case of narcissism as a prototypical example, we argue that personality pathology should not be understood solely as an interpersonal phenomenon but also as a transpersonal one. We propose a new conceptual model which assumes that personality traits can be conceived of as *scripts* which may operate at the level of individuals, dyads, groups, and/or larger social entities. The transpersonal view is fully compatible with trait and interpersonal models, but, beyond that, it provides a framework for understanding variation, regulatory dynamics, and changing phenomena at larger levels. This expanded framework offers a novel lens for understanding the embedding and co-constitution of personality pathology—specifically narcissistic pathology—beyond the individual. Our approach foregrounds the clinical insight that any individual pathology must also be addressed in terms of its social-pathological conditions. Thus, we advocate for a diagnostic and therapeutic model that integrates the intrapersonal, interpersonal, and sociocultural aspects of personality pathology.

The adoption of a dimensional perspective on personality and personality pathology provides a unified basis for the description of different traits within their nonclinical and clinical range. The inclusion of dimensional personality models in the DSM-5 (American Psychiatric Association [APA], 2013) and ICD-11 (World Health Organization, 2019) can be considered a paradigm shift in the description of personality and personality disorders (Hopwood et al., 2013). While these recent developments move the field towards a comprehensive description of personality and personality pathology, it is focused almost exclusively on the individual level, thereby disregarding variation at larger levels. Here, we propose a *transpersonal view*^1^ on personality and argue that personality and personality pathology can be considered as transpersonal phenomena in the way that individuals, dyads, or larger social entities follow predefined sets of rules in terms of *scripts*. We exemplify the model for the prototypical case of narcissism. While the proposed view is fully compatible with individual-centered and interpersonal perspectives on personality, commonly taken in personality and clinical psychology, it can conceptually account for phenomena beyond the person-level (e.g., narcissistic dynamics in dyads or larger social entities) and large-scale variation across cultures and over time.

In personality psychology, one thinks of traits as typical patterns of experience and behavior (Novikova, 2013), which are often implicitly or explicitly assumed to serve a self-regulatory goal at the individual level. For instance, the patterns of experience and behavior associated with the fundamental trait dimension of intro-/extraversion regulate the individual’s need for stimulation (Eysenck, 1967), whereas experience and behavior associated with the more specific trait of narcissism regulate evaluative aspects of the self, particularly one’s self-esteem (Morf & Rhodewalt, 2001). These self-regulatory functions target general needs and motives which concern every individual, not just some. The *extent* to which a particular individual may strive for stimulation, or for self-esteem, may vary. Accordingly, everyone can be allocated on a continuum ranging from introverted to extraverted, or from non-narcissistic to narcissistic. With this dimensional model in mind, we commonly assume that an individual “possesses” a certain level of intro-/extraversion or narcissism.

While the trait approach is well suited to describe individual personality variation, certain phenomena take place at a larger scale and cannot be accounted for by variation in individual differences alone. For instance, it is well documented that romantic relationships can have narcissistic qualities (Nørbi, 2021; for empirical research, see Wurst et al., 2017), which shows that narcissism is at play not just at the individual level but also at the dyadic level. Narcissism is an attribute not just of the involved individuals but also of the relationship *itself* (Jacobs, 2019).

The dyadic level is well researched in personality psychology. This can be seen in the development of research methods and models for dyadic data and social relations analyses (Kenny, 1988, 1996). The concept of narcissism has attracted research interest by interpersonally oriented personality psychologists (e.g., Wilson et al., 2017) using dedicated quantitative methods (e.g., Back et al., 2013; see also Back et al., 2011) since narcissism *requires a counterpart*, and different hypotheses have been put forward regarding complementary and concordant dyadic relationships. These involve a set of “rules” for both partners to stabilize. In other words: both partners follow *one* script (in terms of a set of interaction rules), which stabilizes and regulates the relationship.

Just like a dyad, larger social entities, such as organizations, might also follow a set of rules to stabilize, and to pursue their own narcissistic goals (Wales et al., 2013). And just like in contemporary, individual-centered models of personality and personality pathology, the extent to which larger social entities follow a particular set of rules might be considered dimensional. One might think, for instance, of institutionalized practices of competition and incentive drivenness, or grandiose performance and success goals promoted in organizations that provide a large identification potential particularly for narcissistic agents but also that stand for a specific kind of organizational culture. We extend the view on narcissism by taking a closer look at how it is implemented and sustained *within larger social entities* such as organizations (Jacobs, 2019), and how the intra-, inter-, and trans-personal dimensions of narcissism play out together.

The regulatory processes, which we typically ascribe to the individual, may also take place at a broader cultural level. In quantitative empirical research, the collective narcissism construct was postulated and researched as a form of narcissism which is bound to a collective identity at the national level (De Zavala et al., 2009). Trait-based cross-cultural research indeed shows that there is substantial *variation* not just at the individual level but also between different countries. It was found, for instance, that average levels of narcissism are higher in countries with lower scores on the human development index, meaning that country-level narcissism is an inverse function of country-level health and wellbeing (Jonason et al., 2020).

For the case of narcissism, this suggests that (1) a process-based regulatory model can be established beyond the person level, (2) there is significant variation beyond the person level, and (3) this variation is significantly related to relevant variables at a larger level (such as country-level health and wellbeing; Jonason et al., 2020). Narcissism at a larger scale thus meets criteria that we would commonly stipulate for a trait, but does so beyond the individual level, at the level of dyads, organizations, or cultures. We thus propose that personality may be regarded from a *transpersonal perspective*, with the key assumption being that recurrent patterns of experience and behavior may be seen as *scripts* which are situated *across individuals* (see Figures 1 and 2). The extent to which an individual, dyad, group, or culture acts in accordance with a script—for instance a narcissistic script—is thought to be determined by their respective susceptibility. This transpersonal view is fully compatible with trait models, but beyond that, it can describe regulatory dynamics beyond the person-level variation across cultures and times.

**Figure 1.**
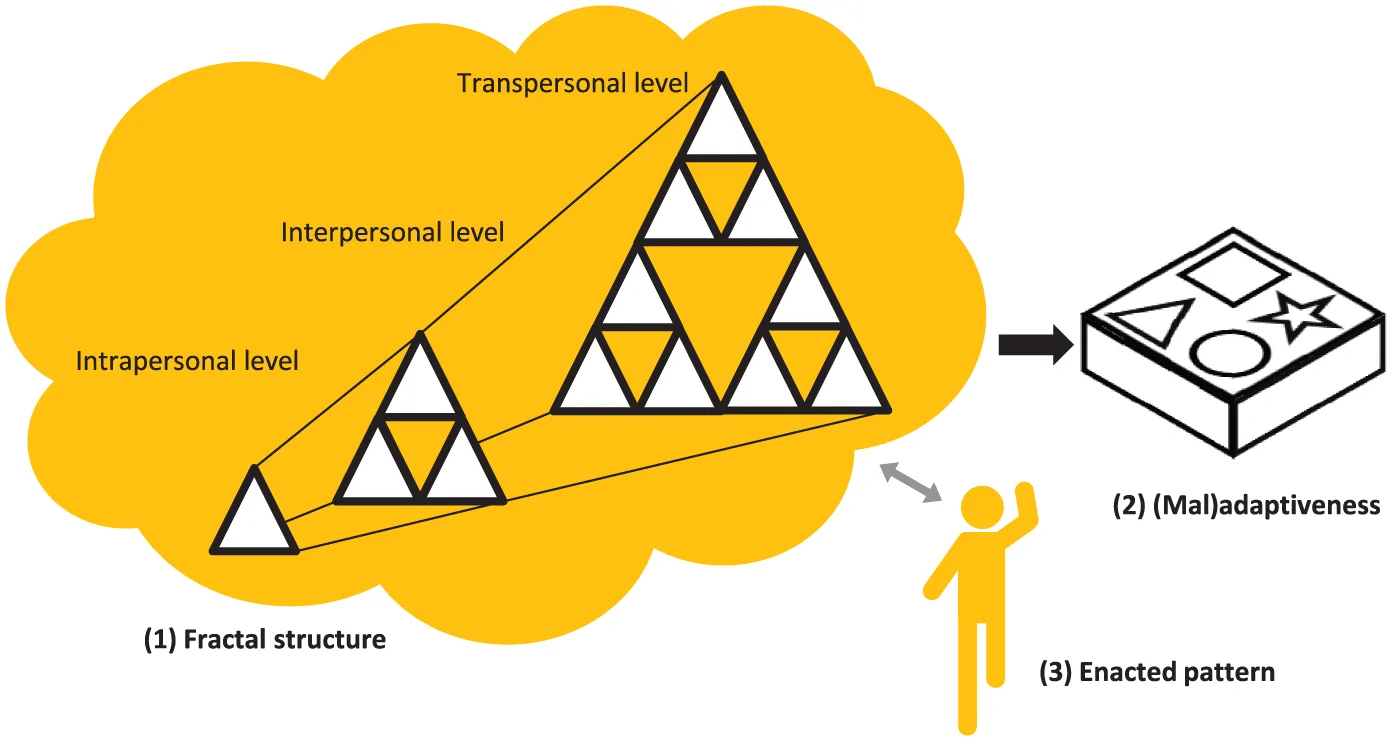
Extensions of the script concept. In the transpersonal model, scripts (1) have a fractal structure that repeats itself on the intra-, inter-, and transpersonal level; (2) may be adaptive or maladaptive with respect to specific environmental conditions; and (3) are being enacted with persons as their carriers, like a dancer “incorporating” a dance script.

**Figure 2.**
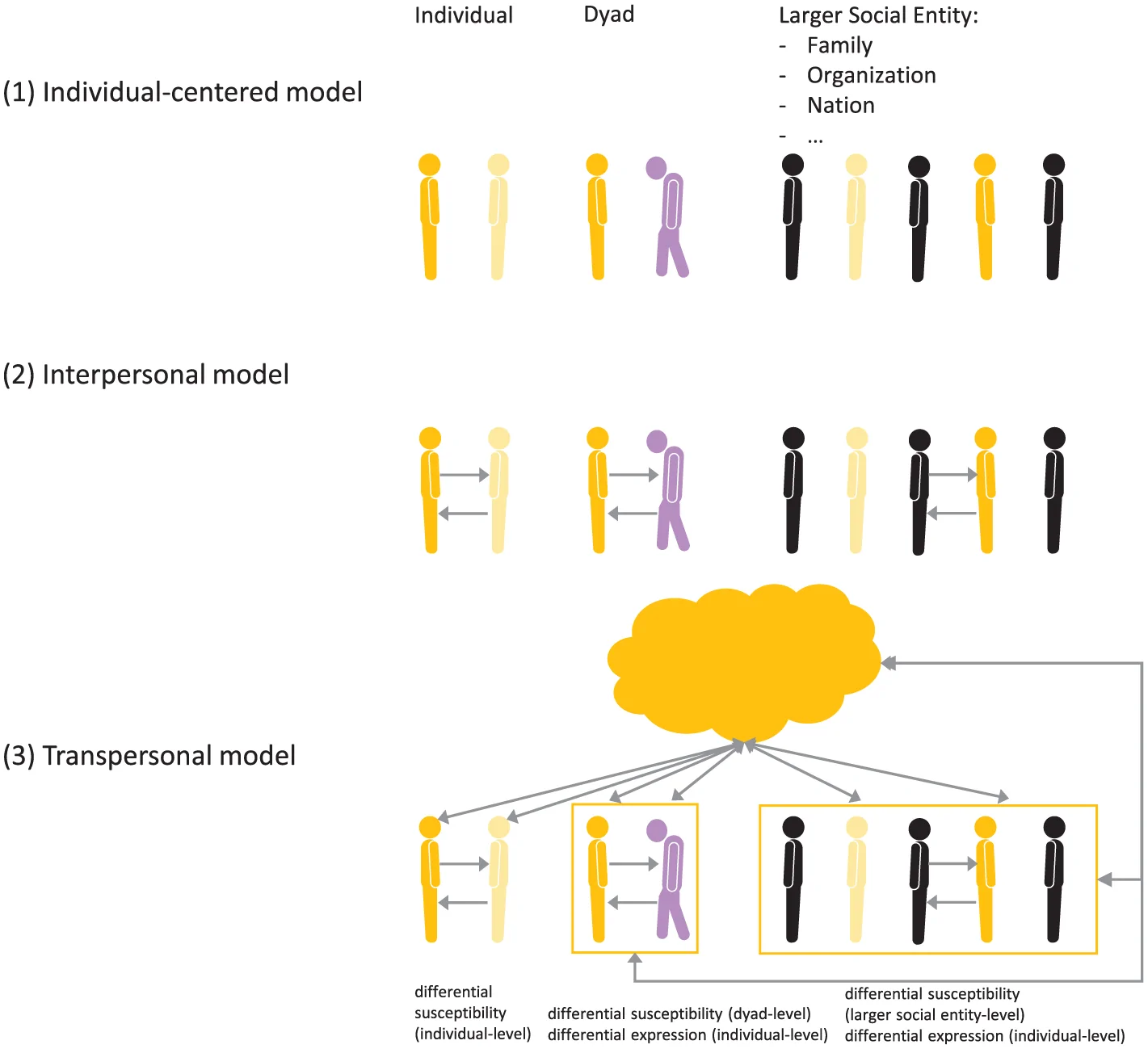
Schematic illustration of individual-centered (top row), interpersonal (middle row), and transpersonal (bottom row) models of personality. In the transpersonal model, observable individual differences in experience and behavior can be conceptualized in terms of scripts which are being enacted by an individual (left panel), a dyad (middle panel), or a larger social entity (right panel). The degree to which an individual, dyad, or larger social entity enacts a certain script depends upon its susceptibility to the respective script, which is situated at the respective level of analysis. Color codes denote differential expressions, in terms of different individual-level phenotypes, corresponding to one script (e.g., narcissistic grandiosity [yellow] and vulnerability [purple]).

In the following, we will briefly review the central assumptions of individual-centered and interpersonal models of personality, highlight features and assumptions which are of relevance to the present work, and discuss their current applications to narcissism. We then present a transpersonal view as a possible extension and demonstrate its feasibility using examples from the literature on narcissism and other clinically relevant personality characteristics. Finally, we provide ideas for first steps towards its operationalization and further development.

## Existing models/paradigms in personality psychology: From the individual to the interaction

### Individual-Centered model

*Trait theory* has emerged in the past century and is the currently predominant paradigm in personality psychology (e.g., Rauthmann, 2017), now also including the description of personality pathology (e.g., World Health Organization, 2019). It assumes that an individual’s personality can be described by specific combinations of traits, whereby a trait is “any distinguishable, relatively enduring way in which one individual differs from another” (Guilford, 1959, p. 6). A central assumption of trait theory is that a trait is a *feature* of the individual (while specific behaviors are described in adverbial terms, traits are *adjectival* terms; Carr & Kingsbury, 1938). Following a nomothetic perspective, every individual may be described with the same individual differences construct, but at different levels of an underlying distribution. From a current understanding, the individual trait level can also be regarded as the set-point of an individual state distribution (Fleeson & Jayawickreme, 2015).

Contemporary trait models—most notably the Five-Factor Model (FFM), including its derivates and extensions—commonly make further assumptions on the nature of traits, some of which are of direct relevance to the model proposed below: traits (1) differ in whether they describe narrow (specific) or broad (abstract) aspects of experience and behavior; (2) are stable over time but display some change over the life course and some malleability; and (3) can be assessed in the same way in different groups of individuals.

(1) Some traits are more specific than others, for instance “enjoy parties” is a more specific trait than “enthusiastic about social contact” or “extraverted” (cf. DeYoung et al., 2007). This conforms to the view that personality can be described in a *hierarchical manner* at different levels of abstraction, whereby more narrow/specific traits at lower levels are bundled into more broad/abstract ones at higher levels of the hierarchy. While specific traits are well suited to predict specific behavior in similar situations, they might not predict specific behavior in other situations. For instance, “enjoy parties” might predict “meet friends” well but might be only loosely related to “take the lead.” Both, however, can be subsumed under the broad trait of extraversion (DeYoung et al., 2007). Broad traits, conversely, do not have as high predictive power as specific traits, but can be applied to a variety of situations (bandwidth-fidelity-tradeoff; Cronbach & Gleser, 1957).
(2) While traits are per definition relatively stable, personality can and does change over the course of a lifetime (e.g., Damian et al., 2019), after life events (Haehner et al., 2023; Van Scheppingen et al., 2016), or during therapy (Nguyen et al., 2020). Research on personality change commonly investigates whether a trait becomes more or less pronounced over time, or following events or interventions, that is, if the trait increases or decreases. For instance, the level of agreeableness increases after transitioning into parenthood (Van Scheppingen et al., 2016). What is not commonly investigated is whether trait expressions might change *not only in quantity but also in quality*. It might be the case that psychotherapy, for instance, does not impact or only mildly impacts someone’s overall level of extraversion (e.g., Nguyen et al., 2020), but it might substantially impact *the way* someone seeks out and cultivates social contact. Similarly, clinical observations show that so-called “hysterical” conversion symptoms (e.g., psychogenic neurological dysfunctions) have largely disappeared in western cultures in the past century but can still be observed in non-western cultures, which can be attributed to different culturally shaped *ways* of expressing psychological distress (e.g., Mentzos, 2015). While systematic empirical research on these phenomena is limited, the model we propose here aims to account for this qualitative variation, providing a possible framework for future systematic investigations.
(3) Related to the question of qualitative differences in traits, personality research has long been interested in the *generality* of traits. Earlier research on the FFM postulated that the “Big Five” are “universal” traits, since studies across countries and cultures consistently yielded evidence for the same five factors (e.g., McCrae & Costa, 1997). These studies, however, compared solutions from exploratory factor analyses to each other on a largely descriptive basis. More recent tests using confirmatory factor analyses come to the conclusion that personality traits, as assessed by FFM inventories, cannot be regarded as invariant, particularly not across cultures (whereas higher degrees of invariance are found for sex or age; Dong & Dumas, 2020). It thus seems that personality might not be as (culturally) universal as initially assumed; particularly not when looking into more fine-grained tests of invariance probing the equivalence of items (i.e., specific/narrow behaviors) across cultures. While the observed differences might be considered a “lack of invariance” from a psychometric perspective, they might also “reflect valid differences in personality traits rather than measurement artifacts” (Dong & Dumas, 2020, p. 7), which again underscores the importance of qualitative differences in experience and behavior.

To sum up: the individual-centered model postulates that individual differences in experience and behavior can be described in terms of traits, which reflect stable individual characteristics. Traits (1) are hierarchically organized, (2) develop over the lifespan, and (3) show some level of invariance across demographic variables but considerable cultural variance.

The trait perspective on narcissism has converged in a widely accepted consensus (cf. Wright & Edershile, 2018). Trait narcissism is characterized by interindividual variation in grandiose aspects—dominance and assertiveness along with an inflated self—and variation in vulnerable aspects—sensitivity and reactivity along with a fragile self-esteem (Pincus & Lukowitsky, 2010; Weiss et al., 2019). These grandiose and vulnerable aspects can be parsed into agentic, antagonistic, and neurotic aspects within the FFM (Back, 2018), which each go along with discernible self-regulatory dynamics, nomological networks, and relations to indicators of psychological functioning and psychopathology. We will highlight how grandiose and vulnerable aspects of narcissism might interact not only at the personal level (e.g., Edershile & Wright, 2021) but also at the interpersonal level.

### Interpersonal model

While traits can account for a significant amount of variance in important dimensions of individual people’s lives, they also leave significant amounts of variance unexplained (e.g., Lavi et al., 2022; Roberts et al., 2007). One reason for this is that personality is always situated within a broader, particularly social context. The interpersonal (interactionist) paradigm of personality psychology has emphasized the interplay of individual experience and behavior with situational characteristics (Kenrick & Funder, 1988), whereby empirical studies show that the relative contributions of personal and situational factors are about equal in magnitude (Richard et al., 2003).

Personality psychology has put forward several models with varying degrees of formalization that seek to describe interactions between individuals and their (social) surroundings. These emphasize different aspects of interactions, such as social roles (neo-socio-analytic-model; Roberts & Wood, 2006), interpersonal perceptions in social interactions (e.g., Back et al., 2013), or co-development of individuals in dyadic relationships (e.g., Cook & Kenny, 2005). The latter of these models have yielded not only new theoretical perspectives but also new research methods which are suited to investigate the proposed effects at an operational level (e.g., Back et al., 2011).

As one prominent example, the interpersonal circumplex (e.g., Kiesler, 1983; Leary, 1957), describes individual experience and behavior on the axes of dominance and affiliation (also referred to as agency and communion). While these do have trait-like characteristics in terms of individual set-points, they also show substantial variation between different interactions, depending on interaction partners. One assumption is that of complementarity, that is, assertive behavior of one interaction partner should be accompanied by submissive behavior of the other (e.g., Tracey et al., 2001), which has also been confirmed empirically (Markey et al., 2003; Sadler et al., 2009). Going beyond trait approaches, the circumplex model thus exemplifies that specific experience and behavior can be considered an *emerging characteristic* of a particular situation, which cannot be captured by individual-centered models. It allows description of interpersonal behavior not only at the level of a particular interaction but also in terms of dynamic, moment-to-moment changes within this interaction (Hopwood, 2018; Hopwood et al., 2013) that may follow sets of *rules* (such as complementarity, in the circumplex example).

Narcissism has been described from an interpersonal, process-based perspective in models such as the narcissistic admiration and rivalry concept (NARC; Back, 2018; Back et al., 2013) or the dynamic self-regulatory processing model (Morf & Rhodewalt, 2001). Going beyond trait models, these models require at least two people to describe narcissistic personality dynamics, making them *inherently interpersonal*. Still, the self-regulatory strategies described in the models are regarded as traits and thus as features of the individual. In the model outlined here, we propose that regulatory dynamics might be understood as *scripts* rather than traits.

### Scripts as units of analysis in interpersonal behavior, and conceptual extensions

In psychology, a script is commonly defined in terms of a body of knowledge about a sequence of events, frequently conceptualized in terms of situationally “appropriate” behavior (cf. Albarracin et al., 2021), therefore “embodying knowledge of stereotyped event sequences” (Abelson, 1981, p. 15). A frequently used example is the knowledge about the series of events taking place when *dining out* at a restaurant: wait to be seated, check the menu, order wine, order food, eat, etc.

Albarracin et al. (2021) propose that the different uses of the script concept in behavioral and social sciences may be organized along two dimensions: (1) internalist versus externalist readings of the script, and (2) weak versus strong readings of the script. (1) *Internalist* reading means that a script is thought of as a cognitive structure which is situated *within an individual*, like the trait concept. *Externalist* readings see a script as “a set of highly codified practices, norms, standards, beliefs, linguistics practices, and rules that make up an institution” (Albarracin et al., 2021, p. 2), thus addressing shared knowledge *within a dyad, or larger social entities such as specific groups or cultures* (Clark & Brennan, 1991). (2) The distinction between *weak* and *strong* readings of the script concerns the extent to which a script is structured and follows a clear sequential order (strong script) or is a rather loose cluster “of associations or contingencies related to specific events” (Albarracin et al., 2021, p. 5).

Demorest (1995) suggested that *personal scripts*, in terms of individual patterns of motives, emotions, and actions of the self and others, may serve as units of analysis for the study of personality. As Demorest argues, like a trait is a personality description from an observer perspective, a personal script is a personality description from an agential perspective. Personal scripts can be of weak or strong nature, but—according to the taxonomy introduced above—they are always internal scripts in that they concern one individual (Demorest, 1995).

Starting from both internalist and externalist readings of the weak script concept, we extend the view on scripts to make it futile for our transpersonal model of personality and the idea of transpersonal narcissism. We propose adding the following features (see Figure 1) to the above-mentioned ones for further conceptualization of scripts:

(1) *Fractal structure*: We have already stated that personality can be described in a hierarchical manner at different abstraction levels, whereby more narrow/specific traits at lower levels are bundled into more broad/abstract ones at higher levels of the hierarchy. Consequently, we emphasize with respect to the *conceptual structure of scripts* three structural relata or dimensions: the intra-, inter-, -and transpersonal levels, according to which one can describe if the script is *enacted*^2^ by individuals, dyads/groups, and larger social entities (such as organizations), respectively. Viewed top-down/bottom-up, each level reflects the more general—in addition to the level-specific—script criteria. In consequence, we have single levels of description, but also a fractal structure or “Matryoshka effect” of scripts given with the three structural relata. This allows us to differentiate the specific functionality and symptomatic phenomenal realization of the script for all three levels and to make transparent *how the core structure repeats itself* on the next respective level (see Figure 1).

 Within our model, each level of script expression can be analyzed distinctively, but also with respect to its specific interdependency and interrelatedness to the respective next level: grandiosity in narcissism, for instance, is associated with the idea of thinking of oneself as being special and entitled to special privileges (*intrapersonal, personal level, micro level*), which may “fit” the dynamics of dyadic relationships (*interpersonal, dyadic/interactive, meso level*), or the organizational level of larger social entities (*transpersonal, institutional, macro level*), as it has been pointed out with analyses of narcissistic organizations (e.g., by Duchon & Burns, 2008; Duchon & Drake, 2009) or collective narcissism (De Zavala et al., 2009). Moreover, how a script is enacted depends on the respective dyadic or group-specific (i.e., institutional-organizational) features: grandiosity might be expressed by entitled behavior, but this implies its interpretation as “entitled” against the background of certain shared social, cultural, or group-specific norms or values that are predetermining how a trait is assessed in a certain way (e.g., as expression of healthy self-worth versus entitlement [see also Kaufman & Jauk, 2020]).

(2) *(Mal)adaptiveness*: Scripts allow agents to adjust to environmental conditions. For instance, the “dining out” script allows adjustment to the “restaurant” environment at the individual level (one person of a couple having dinner), the dyadic level (the couple as such), and the larger social level (the couple, the waiter, etc.). In each environment, agents can predict, and thus anticipate and infer, what is situationally required by understanding the meaning of social interactions and situational cues. Provided with such applicable knowledge, scripts enable adaptation to situational conditions (see Figure 1). Recognition of action alternatives, or changes of environmental, group-specific, or institutional conditions, often come along with a change of script. Scripts are thus *constitutive* for certain modes of enaction, but always are equally dependent on specific (environmental, group, or organizational) conditions. The realization potential, thus mostly also the adaptivity, is limited when scripts cannot be shared as joint patterns of enaction. If they are, for instance, simply not properly understood by agents, that is, they literally make no sense in processes of (joint) “scripted” sense-making processes (for the role of scripts in psychological maladjustment, see also Demorest, 2013).

 Scripts therein also serve as “containers” for all different kinds of sequences of experience and behavior. Not only is each single expression of the script a situational realization or actualization of the script, but single action sequences appear as punctually (in)consistent or structurally (in)coherent with it. To put it differently: certain forms of enaction can(not) be consistently embedded as *script-typical realizations* or can(not) be “contained” by the script. As an example, the dining-out script does not include starting a quarrel with the waiter while ordering drinks. Though this may be realized within the broad scope of the script, it cannot consistently be seen as integral to the dining-out script itself. From that, we can infer for narcissism that it can be further analyzed with respect to the *situational and group-specific conditions that make it more or less adaptive*.

(3) *Scripts as enacted patterns*: Scripts are always both constitutive for and constituted by social practice (i.e., the way we do something). With this, one can focus on how particular scripts not only become a part of but are *the* superordinate enactive pattern of a social entity (see Figure 1). The script “works through” the individuals who are its carriers: it “uses” people and organizational structures as realizers, such as viruses use hosts to spread, and thereby implements itself and shapes social practice. Inasmuch as scripts are interpersonally *effective* patterns, they are (pre)determining the dynamics and actions of interactants in a specific situation. Put differently: a script always comes with an “interactional” offer, that is, if agents are susceptible and share it, it becomes (re-)enacted and thus implements as a perpetuating pattern that shapes the dynamic modes of (inter)action of individuals, dyads, or larger social entities. Individuals, dyads, or groups such as organizations can be analyzed with respect to the repertoire of scripts that impregnate their daily praxis or life-forms. Scripts become corpo*realized* in a literal sense: we incorporate and corporealize them through and by (re)enacting them.

 Like a dancer “incorporates” and enacts the dance script by numerous repetitions in exercise, which then impregnates the body (becomes corporealized) and allows the dancer to perform, narcissism can be a shared pattern of literally corporate agents, such as groups or organizations: it shapes as incorporated social practice how people treat each other and “sets the tone” for interaction on the corporate level of agency. Scripts thus shape the reality of individuals, dyads, or organizations and “materialize” in the respective praxis forms, which structure social entities. They are culturally shared and thus also transferred among specific groups/collectives. Therein, they are playing a significant role in the transfer of (group-)specific knowledge, skills, and other practice forms (e.g., memories such as *collective trauma*; Chentsova-Dutton & Maercker, 2019).

 The script as a pattern is the conceptual background for further exploring how narcissism negatively interferes with the reproduction dynamics of social recognition relations on an inter- and transpersonal level of social functioning (Jacobs, 2022). More specifically, it sheds light, for instance, on narcissistic *defense*. Mentzos (1988) has developed an influential theory of interpersonally organized defense. Individuals or groups usually have the adequate means to meet the demands that a certain organization or even culture places on them. The more people experience organizational structures as “matching” their individual needs or success goals, the more the script can establish also on an institutional level of larger social entities. If one sees the structure of individual mental functioning and organizational functional dynamics as structurally related, defense works also in the form of institutionalized (in our model: transpersonal) defense. Think, for example, of authorities denying accusations of racism within their own structures or companies engaging in “greenwashing” to avoid having to acknowledge environmental problems. In this case of transpersonal defense, mechanisms such as denial, rationalization, or projection serve to preserve the institutional self-image as a moral authority. In this way, an organization immunizes itself against internal and/or external criticism that seeks to identify these shortcomings and the defenses themselves. Scripts as shared patterns may explain why not only individuals or dyads but also organizations become functionally susceptible to narcissism.

To sum up, both behavioral and social sciences have made use of the script concept, but to our knowledge there have been no attempts to conceptualize scripts as concerning both the individual level (internalist reading) and the interpersonal level of (sub-)cultural groups (externalist reading). Just as traits are conceptualized to be hierarchically structured, we conceptualize scripts as having a *fractal structure*, whereby cornerstones of the script can be found at different levels. Scripts may further vary in their *(mal)adaptiveness* and reflect *shared patterns* in terms of social practices. The strength of our model lies in its application to contexts where there is substantial variation in the way motivational goals are satisfied, which we think is the case in narcissism: scripts become intersubjectively and transpersonally effective in and through social praxis. To put it in a phrase: *You never “narc” alone!*

## A transpersonal model of personality, exemplified for the case of narcissism

Figure 2 presents the cornerstones of the transpersonal model. Observable individual differences—usually described in terms of traits—can be conceptualized in terms of scripts which are being enacted by an individual (left panel), a dyad (middle panel), or a larger social entity (right panel). As an analogy, scripts, in this model, might be conceived of as viruses, which cannot reproduce on their own but contain information that affects their hosts in ways that enable their reproduction (Knipe & Howley, 2013). The degree to which an individual, dyad, or larger social entity enacts a certain script depends upon its *susceptibility* to the respective script, which is situated at the respective level of analysis (i.e., just as an individual may be susceptible to enacting a narcissistic script, dyads or larger social entities may be susceptible to enacting it).

The *trans*-personality particularly refers to narcissism as having the potential to “spread” among or expand significantly within dyads, or larger social entities such as groups and organizations, which depends on the susceptibility of individuals but also on the susceptibility of the larger social entity. Narcissism literally can become institutionalized within larger social entities and therein clearly supervenes the individual trait-narcissism view. With respect to its genesis, one would not only refer to its genetics (e.g., see Luo & Cai, 2018; Luo et al., 2014) but, moreover, can ask for specific *social and cultural—“organizational”—conditions* (see also Kohut, 1966) according to which it emerges, is maintained, and is acquired within and through the dynamics of dyads and larger social entities.

Turning back to the transpersonal model, we propose that the script has a *fractal structure*, that is, it provides prototypical sequences of experiential and behavioral patterns not just for one individual but also for dyads and larger social entities (see above and Figure 1). The extent to which a certain script is adaptive or maladaptive, “healthy” or pathological, thus, can be determined at the highest level (e.g., a dyad or group), but affects the lower levels (e.g., an individual). This introduces a further level of complexity, namely that of *differential expression* in terms of different individual-level phenotypes corresponding to one script. As an example, a dyad might be composed of one individual following a grandiose-narcissistic script (displayed in yellow in Figure 2), and one individual following a vulnerable-narcissistic script (displayed in purple). Moreover, even more complex differential expressions might be observed at the level of larger social entities.

Taken together, the transpersonal model thus extends the interpersonal level of description since emergent characteristics can become manifest also in organizationally broader structures than dyads. Experiential and behavioral patterns are always embedded in a way that “uses” the individual as a carrier: narcissism is enacted within the horizonal scope of certain dyads, groups, or societies. These (pre)determine not only its particular expression but also to what extent the script can “anchor in” within a dyad, group, organization, or culture. We therefore use the script concept to explain how a certain experiential and behavioral pattern is carried through and gets “replicated” by its enaction in terms of *cultural transmission* (e.g., Distel et al., 2009). Additionally, scripts are assessed as being (mal)adaptive at the level of intra-, inter-, and transpersonal enaction, respectively, but scripts might also change over the course of time, or across different cultural contexts (e.g., Ryder et al., 2014). For instance, it might be the case that agentic (i.e., socially dominant) forms of narcissism are becoming less socially accepted, and narcissistic strivings are channeled into more socially accepted, communal (i.e., seemingly prosocial) expressions (cf. Gebauer et al., 2012). However, to our knowledge, no study to date has tested such qualitative changes. The framework presented here might stimulate such research.

Regarding the replication of scripts, one may draw an analogy to a virus: a script, like a virus, uses a host to spread, while its successful implementation is dependent on its being (re-)enacted (see the second section). It thereby can alter dyadic, group-specific, or organizational dynamics metaphorically spoken “in its favor” (like a virus resides in its host and benefits as long as the hosts spread its information). Narcissism may spread also among larger social entities, in such a way that it not only affects the respective individual agents (of a dyad, group) but becomes engrained into the organizational structure.

But what determines the flourishing or restricts the spreading of a script? We propose that this is due to a *differential susceptibility* to enacting it. This susceptibility may depend upon different factors, but as a necessary condition it requires a *motivational state* (of individuals, dyads, groups, organizations) which is congruent with the functionality of the script. In the case of narcissism, such a state can be seen on an intrapersonal level primarily in the (conscious but also unconsciously present) motivation to fuel one’s self-esteem (Back, 2018; Morf & Rhodewalt, 2001). This motivational state might be consistent with scripts containing strategies for agentic self-enhancement or antagonistic self-protection (Back, 2018) but might not be consistent with scripts oriented towards cooperative behavior, for instance.

Consider, for example, a group of coworkers playing a bowling game after work. Person A, with stable self-esteem, might enjoy the time spent with their coworkers and the competition no matter if they win or lose, and thus have *low susceptibility* to enact a narcissistic script in this situation. For person B, who is motivated to fuel their self-esteem, the situation might have high self-relevance, and person B might have *high susceptibility* to enact a narcissistic script, the target of which is to self-enhance after own wins (“see how I did this?”—agentic strategy) or self-protect after own loses (“you must have had good luck to defeat me”—antagonistic strategy; Back, 2018).

Similarly, a *group of individuals* might, together, have low or high susceptibility to enact the narcissistic script. Company A might, for instance, be well known for producing small series of high-quality bicycles for which there has always been a sufficient market. Company members feel confident about their product, and company A does not have the need for self-enhancing or self-protective strategies, making it a *low susceptibility* organization. Company B also produces bicycles, which are of rather low quality but also very cheap. Since the company doesn’t earn much with single bicycles being sold, the company’s overall strategy is to increase profit by increasing sales. Compared to company A, company B thus is in a motivational state, which goes along with a *high susceptibility* to employ narcissistic scripts, such as for instance dishonestly exaggerating the product’s features or strategically discrediting its competitors (Böckler et al., 2017).

While the group-level example refers to the behavior of the two companies *as organizations* in relation to other groups/organizations, the enactment of the script requires that individuals within the organization take certain roles. In company B, for instance, some individuals within the organization might have a greater urge to comply with a narcissistic script than others. The managers responsible for increasing production and sales, for instance, might be central to the organizational implementation, sustaining, and maintenance of the narcissistic script, but this may be of less concern to the worker who manufactures the product. Thus, while there is differential susceptibility at the level of the organization, there is *differential expression* of narcissistic scripts at the individual level within the same organization.

Going one step further and assuming that the conditions for production workers are very bad and the workers are being exploited in company B, it might be that the manufacturing worker, complemental to the script of the production manager, displays signs of narcissistic vulnerability (such as feelings of insufficiency) rather than grandiosity. The concept of *differential expression* thus may describe not only quantitative differences in the expression of one form of narcissism (grandiosity) but also *qualitative* differences in terms of complemental roles (vulnerability).

The susceptibility parameter offers a seamless transition to trait models, in that it can be thought of as the *likelihood to enact a certain pattern of experience and behavior*. Though the proposed model is thus compatible with trait accounts, the difference to a trait perspective is that we consider patterns of experience and behavior, in terms of manifestations as enacted scripts, as *transpersonal in nature rather than situated within the person*. This difference is important because it can account for phenomena such as cultural differences or trait changes over time. Given there would be no variation in patterns of experience and behavior across different cultures or time, we acknowledge that the transpersonal model and the trait model would reach the same predictions, and the trait model should be preferred as the more parsimonious model.

The transpersonal model of personality (pathology) refers to the idea that script enaction is inextricably intertwined with *institutional and cultural organization*. This builds a bridge to culture (psychoanalytic) theory and reminds that “individual psychology is at the same time social psychology as well” (Freud, 1921/1955, p. 69).

One question is, then, how the *functional effects of the narcissistic script* can be related to the complex functionality and dynamics of larger social entities, that is how the script “interferes” with the basic functional dynamics of dyads, groups such as organizations, or even organizational networks (Jacobs, 2019).

Generally, a larger social entity can be considered a (potentially) disordered entity, if the organization is in a dysfunctional state that produces non-trivial harm to either their members or other organizations. Such harms not only affect (in a health-impairing way) real people but can cause non-trivial damage to an organization as a functional whole (Jacobs, 2022). We believe that such harmful dysfunctions can be operationalized and measured, e.g., with respect to *negative long-term effects* that turn out to be the result of narcissistic goal attainment (see as described by, e.g., Lubit, 2002). The dysfunctionality, together with non-trivial harms that are caused by this dysfunction, determines the “pathogen” or even pathological status of an organization.

Consequently, it might sometimes be necessary to make these script dynamics transparent to initiate change. In the same way individuals and couples can seek out therapeutic intervention, so too larger social entities might need interventions to deal with the effects of the script (see also Kets de Vries, 2004). We suggest that the narcissistic script plays a significant role for describing, for instance, phenomena of “immunization” strategies of narcissistic groups or organizations against critique, which can be thematized as a signature of the ethical failure of institutions (Clarke & Dean, 2014; Jaeggi, 2009).

Narcissistic leadership within organizations (e.g., Rosenthal & Pittinsky, 2006) is a real-life example of the harmful dysfunctional change induced by a shared script. Here, the so-called “France Telecom suicides” (e.g., Van Overstraeten, 2022) can be seen as an instance of suicide committed by employees because of a strategy change (a goal change) at the company. The management methods that had been implemented degraded working conditions with the aim of making people voluntarily leave, in order not to have to pay compensation. This form of reification can be seen as a typical example of narcissistic abuse.

An environment that is less susceptible (i.e., less critically sensitive to strategies of narcissistic manipulation) might stay rather unresponsive to these strategies; the script is incompatible with what is intersubjectively or transpersonally (e.g., sub-culturally or at the normative level of values or beliefs) shared within a certain dyad, group, or organization. This allows thematizing scripts in their *fitting relation* with the (organizational) ethos or rules of groups or organizations, which is implemented in terms of *differential susceptibility* in our model (see above).

## First steps towards the operationalization of transpersonal phenomena

The study of transpersonal phenomena might take place in three stages in a mixed methods design: (1) areas for the study of transpersonal phenomena might be identified using quantitative methods by investigating variance beyond the person level; (2) relevant scripts might be described using narrative, qualitative accounts; and (3) on the basis of these quantitative and qualitative findings, hypotheses might be formulated and tested quantitatively.

(1) As outlined above, individual differences research has brought up methods which may capture variance in psychological variables beyond the person level. For dyadic data, this may be accomplished using methods such as actor-partner-interdependence models (Kenny, 1996) or social relations models (Kenny, 1988), which allow, amongst other things, to study interactive, reciprocal effects of relationships. At the level of larger social entities, general *multilevel models* may be used to study variance related to groups such as families, organizations, or countries. These allow decomposition of variance to investigate the percentages which may be attributed to the individual level or the larger, cluster level. Clusters of transpersonal relevance might be identified by significant variance at the cluster level, indicating that individuals within a cluster share a certain degree of similarity (or also complementarity).

 While multilevel models are being routinely employed in some fields of psychological research (e.g., in pedagogical psychology), research on personality and personality pathology does not commonly investigate variance beyond the person level. This might, however, yield important insights. Going back to the bicycle companies example, it might be the case that there is significant variance not only in (individual) employee narcissism levels^3^ *between* the companies but also in measures of job satisfaction or goal orientation, which relates to *one* company-level variable, namely the *transpersonal script*. This script, however, is unknown at this stage, which leads to the next point.

(2) Once a relevant cluster level has been identified, the next stage could be to describe the script enacted within this cluster by identifying contingencies affecting individual experience and behavior. This may be accomplished by employing narrative methods, that is by interviewing individuals of the respective cluster about significant experiences (Demorest, 1995; Thorne, 1995) to gain an understanding of *how* certain patterns of experience and behavior are organized. Narrations might then be coded using relevant categories. Elaborate coding schemes have been proposed for analyzing scripts centering around one individual (e.g., Demorest et al., 2012), and these might be directly used to study similarity in patterns of experience and behavior within a cluster.

 In the bicycle companies example, an analysis might show that company A employees might feel confident about their work and products, experiencing no particular pressure or distress, whereas employees from company B might report feelings of distress. Organizational leaders from company A might report pursuing qualitative goals, whereas leaders from company B might report pursuing quantitative goals. While simplified, this example shows that the clustering method allows study of *complemental* aspects of scripts within a cluster (here: employees versus leaders). While, admittedly, a sufficiently exhaustive description of such complemental aspects might be challenging for larger social entities, it might well be suited for the study of scripts within dyads or in small groups such as families.

 Once the analysis has yielded an abstraction of the individual patterns of experience and behavior (an “up-translation” to the level of scripts; Thorne, 1995), cornerstones of this abstract script might be assessed in terms of individual experience and behavior, and individual agreement with the script, which leads to the last point.

(3) One might test the validity of a script using quantitative indicators of relevant markers such as psychological traits or state variables, or also directly measure individuals’ agreement with up-translated aspects of the script. The same multilevel framework as in stage 1 might be used, but hypotheses tests are no longer limited to differences in the mean structure but can be extended to the correlational structure, to assess contingencies between different script aspects. For instance, the hypothesis might be tested that organizational maximization of profit correlates with employees’ feelings of distress.

 Finally, the same logic can be applied to trait measures themselves, as this is the case in cross-cultural research. If one hypothesizes that narcissism scripts differ between cultural groups, this can be assessed by evaluating the structure of narcissistic traits between these using multigroup factor analyses. For instance, comparing two countries with a predominately independent (Germany) versus interdependent (Japan) orientation, it was found that narcissistic vulnerability has a similar structure in both cultures, whereas grandiosity has a different structure. Differences were most pronounced for the agentic aspects, which are arguably hardest to reconcile with scripts prevalent in Japan (Jauk et al., 2021). Similar techniques might be applied to changes over time, opening the possibility to study changes in scripts in relation to cultural changes (e.g., Wetzel et al., 2017).

To sum up, scripts might be assessed in an iterative process within a mixed methods cycle of quantitative and qualitative research methods. While these are frequently applied, the novel aspect is to use them for advancing a dynamic understanding of personality beyond traits, and beyond a one-person psychology.

## Implications and future directions

So far, we have discussed the cornerstones of the transpersonal model and the extended script concept. Further important aspects concern the implications and possible extensions of the model. Regarding the model implications, we think that psychotherapy is a prime candidate for a field in which it might make a significant difference whether personality pathology is seen as a “harmful dysfunction” (Wakefield, 1992a, 1992b) that is not (only) an individual-level problem. Many therapeutic accounts have already adopted this view, as evident for instance in the concepts of collusive relationships (Nørbi, 2021), systemic pathology (e.g., Bateson et al., 1956), and transgenerational pathology (Volkan, 2001). The formal models used in personality and clinical psychology, however, lag behind these developments. The aim of our work is to bridge this gap by taking the first step towards a model of personality pathology beyond the individual.

Regarding possible extensions of the model, it might be interesting to see whether the proposed fractal structure can be assumed not only at levels beyond the individual but also within the individual (at the intrapsychic level). It could be hypothesized that internal object relations or core beliefs about interaction patterns also reflect core elements of, for instance, a narcissistic script. Considered within the virus metaphor discussed above, these internal models might reflect the “cells” in which the crucial information is encoded. A promising approach for future work might thus be to trace the proposed fractal structure of scripts from the micro to the macro level and vice versa.

Finally, broadening the scope with a three-level analysis contributes to the interdisciplinary and transdisciplinary dialogue in pathology research. Our transpersonal model can be applied to explain how narcissism becomes further implemented and reenacted within and through broader institutional or collective dynamics. To capture the pathogenic impact, future analyses should focus on the constitutive conditions not only for the prevalence of the script and its group-specific spreading conditions but also for how it precedes and is maintained on a transpersonal level. Moreover, the catalogue of symptoms of narcissism can be enriched. Our description of *distortions of functions of an organization* reveals in the interplay (or congruence) of both organizational “achievements” and in the individual striving of script carriers. Perceiving narcissism as institutional or social pathology allows expansion of the clinical *heuristics* and the *repertoire for diagnosing narcissism* in a transpersonal way (Jacobs, 2019, 2022).

## Conclusion

We proposed a transpersonal model of personality and personality pathology, exemplified in the case of narcissism. We stressed the paradigmatic turn from the individual to the interpersonal view. An interpersonal view opened the conceptualization of personality processes, including narcissism, as scripts. By extending the view on scripts, we aimed to make the script concept futile for our transpersonal model of personality and the idea of transpersonal narcissism: in analogy to viruses, scripts contain information that can be enacted by their carriers. Beyond the individual level, these can also be dyadic structures or larger social entities such as groups, organizations, or cultures. Our takeaway message is that it is necessary to combine psychological and sociocultural perspectives on narcissism to show how individual pathology and social structures are interrelated.

## Notes

1. We use the term transpersonal in the sense of *across persons*, to refer to the conceptual idea that certain phenomena which are commonly considered person characteristics may be understood at the cross-personal level.

2. With “enaction” we refer to a prominent position in the cognitive sciences that argues that the interaction dynamics between an organism (a social entity, such as an individual agent) and its environment constitute cognition. Scripts are modes of meaningful relatedness and are constituted by and constitutive for our active encounters, as they shape the dynamic interaction with the environment.

3. We note that this applies to constellations of *corresponding* traits (in this example, grandiose-narcissistic traits in all employees), whereas constellations of *complemental* traits (grandiose-narcissistic traits in some employees, vulnerable-narcissistic traits in others) might also be relevant.
